# Perspectives of pharmacy employees on an inappropriate use of antimicrobials in Kathmandu, Nepal

**DOI:** 10.1371/journal.pone.0285287

**Published:** 2023-05-03

**Authors:** Nistha Shrestha, Sulochana Manandhar, Nhukesh Maharjan, Devina Twati, Sabina Dongol, Buddha Basnyat, Stephen Baker, Abhilasha Karkey

**Affiliations:** 1 Oxford University Clinical Research Unit, Patan Academy of Health Sciences, Kathmandu, Nepal; 2 Nuffield Department of Medicine, Medical Sciences Division, Centre for Tropical Medicine and Global Health, University of Oxford, Oxford, United Kingdom; 3 Cambridge Institute of Therapeutic Immunology & Infectious Disease (CITIID) Department of Medicine, University of Cambridge, Cambridge, United Kingdom; National University of Singapore, SINGAPORE

## Abstract

**Background:**

Unregulated antimicrobial use is common in both hospital and community settings of low- and middle-income countries (LMICs). However, discrete data regarding the use/misuse of antimicrobials at pharmacies in LMICs are limited. This study was conducted to understand knowledge, attitude, and practice of pharmacy employees on antimicrobial dispensing in Nepal.

**Methods:**

We conducted a cross-sectional survey using a structured questionnaire on 801 pharmacy employees working in community and hospital pharmacies located in Lalitpur metropolitan city (LMC) of Kathmandu, Nepal between April 2017 and March 2019.

**Results:**

A majority (92%) of respondents agreed that demand for non-prescription antimicrobials was common. Asking for prescription before dispensing was ranked as the first preference by majority (69%) of participants. Suspected respiratory tract infection was the most common reason demanding for non-prescription antimicrobials with the highest mean rank of 1.5. Azithromycin was the most commonly prescribed and sold antimicrobial, as reported by 46% and 48% of participants respectively. A majority (87%) of respondents agreed on antimicrobial resistance (AMR) to be a global public health threat; and misuse/overuse of antimicrobials was perceived as the most common cause of AMR with a mean rank of 1.93.

**Conclusion:**

Our study revealed that unfounded dispensing and use of antimicrobials is prevalent among pharmacies in Kathmandu, Nepal. This over reliance on antimicrobials, notably azithromycin, may escalate burden of AMR. We identified several drivers of inappropriate antimicrobial dispensing practice in pharmacies, which will aid public health authorities in addressing these issues. Further studies considering role of other stakeholders, such as doctors, veterinarians, general public, and policy makers are required to obtain a more holistic perspectives on practices of antimicrobial use so to curb the extant AMR crisis.

## Introduction

The global burden of antimicrobial resistance (AMR) is steadily increasing, while the discovery of new antimicrobials has effectively stopped, creating new challenges in therapeutic management of infectious diseases [1]. Therapeutic management of previously readily treatable diseases, such as typhoid fever, is becoming difficult because of rapid emergence of multi-drug resistant variants [2]. It is known that use/misuse of antimicrobials is one of the key factors aiding an accelerated evolution, selection, and spread of antimicrobial resistant bacterial pathogens [1]. Rampant and unregulated use of antimicrobials is a common practice in community and hospital settings in low- and middle-income countries (LMICs) [1,3]. With a high burden of infectious diseases, coupled with poor sanitary infrastructure and poor public hygiene, LMICs, such as Nepal play a major contributory role in the increasing global burden of AMR [3]. However, discrete data on various aspects of antimicrobial use/misuse is seldom reported from such settings.

In Nepal, local community pharmacies often serve as the first point of contact for healthcare seeking individuals. These facilities serve as outlets for purchasing antimicrobials as prescribed by doctors. Additionally, pharmacies are also the preferred channels for obtaining a presumptive diagnosis of health issues and accessing non-prescription antimicrobials either as demanded by the patients, or as advised by pharmacy employees. Therefore, pharmacy employees are arguably the key community stakeholders governing use/misuse of antimicrobials in communities of LMICs. Studies have shown that directly accessing antimicrobials at pharmacies as over-the-counter (OTC) drug offers economic incentive to patients by avoiding extra money, time, and distance that would otherwise be required when undertaking formal health advice in healthcare facilities [4]. Apart from ignorance of patients on consequences of self-medication of antimicrobials, lack of stringently applied regulations have been considered as key drivers of non-prescription antimicrobial usage [4]. Understanding perspectives of pharmacy employees on various aspects of antimicrobial dispensing is thus important to understand the community drivers of AMR in a local setting.

Only few such studies have been reported from Nepal [5]. While earlier studies focused mainly on community pharmacies only, in this study, we aimed to include different types of pharmacies located in urban as well as rural outskirts of Lalitpur district. We performed this study to fulfill existing gaps regarding knowledge, attitude, and practices of hospital and community pharmacies on various aspects of antimicrobial dispensing and use in Kathmandu, Nepal.

## Materials and methods

### Ethical approval

An ethical approval for conducting this study was obtained from Nepal Health Research Council (NHRC) under the registration number of 106/2017, and from Oxford Tropical Research Ethics Committee (OxTREC) under the registration number of 533–17.

### Study tools

The questionnaire used in this study was pretested in a pilot survey in 21 pharmacies within Lalipur Metropolitan City (LMC). Initial survey was designed to assess the domains on characteristics regarding prescription and dispensing of antimicrobials, challenges when dispensing antimicrobials, knowledge on AMR, unethical trade practices, and self-medication. The questionnaire was examined for content validity by experts and tested in the pilot survey. After evaluation of the pilot survey, the questionnaire was edited and finalized for this study. The final questionnaire included the domains on dispensing practices/challenges, infection-specific demand for antimicrobials, characteristics on prescription and sale of antimicrobials, irrational prescribing behavior, perception on AMR, efforts to fight against AMR, and unfair trade practice of antimicrobial agents. Open-ended questions were added where necessary to include any response not covered by close-ended questions. The final questionnaire has been appended as a (S1 Appendix).

### Study site and data collection approach

Data collection was conducted in multiple steps. Firstly, pharmacies located in different wards of LMC were physically mapped. For this, the latest information on registered pharmacies available from Department of Drug Administration (DDA) Nepal was used as a basis. As of DDA data from 2016, there were 747 registered pharmacies in LMC, Kathmandu, Nepal. Based on this data, study enumerators spatially mapped community and hospital pharmacies in different wards of LMC between April 2017 and March 2019.

After locating pharmacies, in the first in-person meeting, study enumerators approached pharmacy employees to briefly explain about the study, and asked for their willingness to participate in the study. A verbal informed consent was taken from the willing participants, and a brief questionnaire on demographic information was filled. This included information on type, location, and GPS coordinates of pharmacy; and education level of employees. Additionally, an appropriate date and time as per convenience of potential participants were also recorded so that main data collection could be scheduled in the next meeting. Accordingly, a second in-person visit was planned by enumerators, during which a written informed consent was taken from the participants willing to participant in the study. The participants were asked questions regarding their knowledge, attitude, and antimicrobial dispensing practices in an interview format based on previously piloted structured questionnaire with a mixture of open- and close-ended questions. The close-ended questions had multiple pre-defined options that the respondents were asked to rank each based on their preference, with rank 1 being the highest preference. When the questionnaire could not be completed in a single sitting, further one or more subsequent visits, as needful, were planned as per convenience of participants. Enumerators conducted data collection with each study participants in at least two in-person meetings to ensure completeness of survey. Responses were recorded on a tablet-based questionnaire accordingly.

### Definition of terminologies used in the questionnaire

The following are definitions of specific terminologies and phrases used in the questionnaire. These definitions were used and elaborated as such by the enumerators during the interview-based data collection process in this study (Table 1).

**Table 1:** Definition of terminologies and phrases used in the study.

Terminology	Definition
Self-medication	Selection and use of antimicrobials by patients visiting pharmacies to treat self-recognized symptoms
Higher-end antimicrobials	Broad-spectrum antimicrobials
Poly-pharmacy	An inappropriate use of antimicrobials arising from simultaneous use of antimicrobials having similar mode of action or belonging to the same antimicrobial class
Irrational prescribing	Illogical approach of prescribing medicine failing to confirm to optimal standards of treatment
Wrong dose	Mistakes arising from miscalculation of a dose of medicine
Asked patient to refer to respective doctor	This inferred the requesting patient to consult with his doctor to receive clarification on prescription
Referred to respective doctor	This inferred to an active initiation from the pharmacy employee himself asking with respective doctor for clarification on a given prescription confusion
**Dispensed after correction**	This meant the pharmacy employee uncovered some mistake in prescription by himself or upon consulting with others, and then dispensed after his conscious correction
**Refused to dispense**	This meant the pharmacy employee was not convinced of the given prescription and thus decided not to dispense
**Sought help with a supervisor**	This inferred to an active initiation from the pharmacy employee by consulting with a someone senior available in the store to clarify any confusions
**Dispensed anyway**	This inferred that the pharmacy employee took no corrective actions and just dispensed despite knowing that the prescription may be incorrect
Poor patient compliance	This inferred to patients’ failing to comply with antimicrobial prescription due to patient and/or pharmacy-led fault resulting into under administration of required antimicrobial course
Uncontrolled over-the-counter sale	This inferred to the misuse of antimicrobials arising from pharmacy or patient’s side leading to an over use of antimicrobials
Poor infection control	Sub-standard disinfection measures specifically in healthcare settings
Inadequate sanitary conditions	Sub-standard hygiene measures in community and hospital settings
Patient counseling for prudent use	Advising patients on appropriate use of antimicrobials before dispensing
Improve patient compliance by extensive counseling	Continued advising specially for regularly visiting patients so as to improve their prescription adherence
Bonus	A monetary benefit provided by pharmaceutical companies to entice sell of their specific products
Free samples	Batches of free medicines provided by pharmaceutical companies
Gifts	Non-monetary items, such as goods or services offered by pharmaceutical companies to influence unethical sell of their specific products
False representation	Falsified antimicrobial agents of sub-standard quality deliberately and deceitfully attempted to pass themselves off as genuine approved ones

### Data analysis

The data entered in a tablet platform was electronically uploaded in a secure database, and was rechecked and verified. Raw data has been appended as (S2 Appendix). At the end of the study, data was exported from the database to an excel worksheet for a statistical analysis. The descriptive statistics of close-ended questions were expressed as percentage (%). For the close-ended questions having up to four options, the results were shown as rank proportion for each option, while for those having more than four options, the results were shown as mean rank. The quantitative variable was calculated as mean ± standard deviation (SD).

## Results

In this study, pharmacy employees from a total of 801 pharmacies in LMC, Kathmandu agreed to partake in the study. During field visits, additional pharmacies not yet updated in DDA-2016 list were uncovered, of which 54 consenting pharmacies were included in the study, totaling 801. Privately-owned community pharmacies were the most prevalent (53%; 426/801), followed by private pharmacies inside hospital premises (27%; 218/801) (Table 2). Relatively, representation of government-owned pharmacies was the least (4%; 29/801). A majority (59%; 472/796) of pharmacy employees had pharmacy-specific qualification and thus were licensed practitioners (Table 2). Nineteen percent (151/801) of respondents mentioned of taking pharmacy-related orientation training. This was a month-long orientation training on basic aspects of pharmacy, provided by authorized organizations in Nepal, the certification of which enabled the trainee to register a pharmacy in Nepal. This course however has been discontinued now.

**Table 2:** Basic demographic information of pharmacy respondents.

Demographics	Frequency	Percent
**Types of pharmacy**		
Private owned	772	96
Pharmacy in community	426	
Pharmacy inside private hospitals	218	
Pharmacy inside private clinics	128	
Government owned	29	4
Pharmacy in community	4	
Pharmacy inside public/ semi-public hospitals	25	
**Education of respondents**		
Pharmacy specific education	472	59
Master of pharmacy	10	
Bachelor of pharmacy	121	
Doctor of pharmacy	2	
Diploma in pharmacy	339	
Health-related education	119	15
Other education	59	7
Orientation training in pharmacy	151	19
**Total**	**801**	

### Antimicrobial dispensing practice and challenges faced

The results on responses of participants on various aspects of antimicrobials dispensing are given in Table 3. Not all respondents answered all questions. Overall response rate for this domain was 89%. A high proportion (92%) of respondents agreed that patients often request for antimicrobials without any medical prescription. To get a tentative measure on a volume of demand for non-prescription antimicrobials, the respondents were also asked about frequency of such demands per day, which was found to be an average of 5.8±5.6, ranging from 0 to 50 orders per day. When asked about their approach on handling requests for non-prescription antimicrobials, 69% of respondent ranked asking for prescription as their first preference, while 63% of respondents ranked dispensing non-prescription antimicrobials after asking questions on clinical presentation of patients as the second preference. Respondents were also asked about the challenges they faced in dispensing of antimicrobials. In terms of the challenges from patient’s aspects, self-medication was ranked as the first hurdle as identified by 58% of respondents. In terms of treatment-specific challenges faced by the respondents, no marked difference in ranking preferences of given options were observed, though poly-pharmacy appeared to be ranked as the first hurdle by 33% of respondents.

**Table 3:** Response of participating pharmacy employees on various questions related to their knowledge, attitude, and practice on antimicrobial dispensing.

Questions	Answer choices	Response (%) or, Mean rank	Rank proportions			
			Rank 1 (%)	Rank 2 (%)	Rank 3 (%)	Rank 4 (%)
***Domain 1*. *Dispensing practice and challenges faced***						
**Do patients ask for antimicrobials without a prescription?**						
	• Yes	92				
**How do you deal with patients asking for non-prescription antimicrobials?**						
	• Ask for prescription		**69**	24	7	-
	• Inquire details about clinical indications before dispensing		36	**63**	1	-
	• Dispense antimicrobials right away		4	15	**81**	-
**What kind of challenges do you face while dispensing antimicrobials?**						
	**Patient-specific challenges**					
	• Self-medication		**58**	21	11	10
	• Patients asking for higher-end antimicrobials		13	**35**	32	20
	• Patients unwilling to be counseled		9	27	31	**33**
	• Less aware and uneducated patients		22	20	25	**33**
	**Treatment-specific challenges**					
	• Poly-pharmacy		**33**	23	30	14
	• Overuse of antimicrobials		25	**38**	25	12
	• Irrational prescribing		25	19	22	**35**
	• Frequent use of higher-end antimicrobials		24	23	22	**31**
***Domain 2*. *Specific demand for antimicrobials***						
**What are the most common complaints of patients for antimicrobial request?**		(mean ranks)				
	• Respiratory tract infections	1.50				
	• Gastro-enteric diseases	2.53				
	• Urinary tract infections	2.88				
	• Skin infections	3.78				
	• Dental problems	4.26				
**What are the top 3 selling broad-spectrum antimicrobials?**						
	• Azithromycin	48				
	• Amoxicillin	22				
	• Cefixime	12				
	• Amoxicillin-clavulanate	7				
	• Others	11				
***Domain 3*. *Irrational prescribing behavior***						
**Have you noticed any irrational prescribing?**						
	• Yes	58				
**If you noticed irrational prescribing, what were they?**						
	• Wrong dose		**48**	44	8	-
	• Poly-pharmacy		44	**48**	8	-
	• Others		41	16	**43**	-
	• Unclear handwriting	81				
	• Mistake in dose frequency	10				
	• Spelling error	6				
	• Others	3				
***Domain 4*. *Tackling irrational prescribing and challenges***						
**When faced with irrational prescribing, how did you deal with?**		(mean ranks)				
	• Asked patient to refer to respective doctor	1.76				
	• Referred to respective doctor	2.41				
	• Dispensed after correction	2.83				
	• Refused to dispense	2.94				
	• Sought help with a supervisor	3.29				
	• Dispensed anyway	3.93				
**When dealing with irrational prescribing, what are the challenges you had to face?**						
	• Uncooperative doctors		**59**	32	9	-
	• Annoyance from patients		49	**50**	1	-
	• Pressure to dispense from employers/ supervisors		16	41	**43**	-
***Domain 5*. *Perception on antimicrobial resistance***						
**Do you consider antimicrobial resistance as a global public health threat?**						
	• Yes	87				
**What kind of activities might trigger/ cause antimicrobial resistance?**		(mean ranks)				
	• Misuse and overuse of antimicrobials	1.93				
	• Poor patient compliance	2.92				
	• Uncontrolled over-the-counter sale of antimicrobials	3.66				
	• Irrational prescribing	4.56				
	• Unregulated antimicrobial marketing	5.31				
	• Poor infection control	5.44				
	• Genetic changes of microorganisms	5.57				
	• Inadequate sanitary conditions	6.32				
***Domain 6*. *Efforts on fight against antimicrobial resistance***						
**What can be done from your part as a pharmacist to control antimicrobial resistance?**		(mean ranks)				
	• Patient counseling for prudent use	2.36				
	• Judicious antimicrobial dispensing	2.44				
	• Improve patient compliance by extensive counseling	2.83				
	• Communicate with doctors for better patient treatment	2.98				
	• Discourage unethical pharmaceutical marketing	4.39				
**Have you ever bought antimicrobials from a pharmacy without prescription?**						
	• Yes	64				
***Domain 7*. *Unfair trade practice of antimicrobial agents***						
**Have you noticed any unfair trade practices in your career?**						
	• Yes	87				
**In which area does the unfair trade practice occur more?**						
	• Both government and private pharmacies	59				
	• Government pharmacies	11				
	• Private pharmacies	30				
**What are common types of unfair trade practices you have noticed in your practice?**						
	• Bonus		**49**	38	12	1
	• Free samples		18	**33**	26	23
	• Gifts		13	27	**45**	15
	• False representation		37	7	15	**41**
**Do you give advice to patients to purchase a particular brand of medicine?**						
	• Always	16				
	• Never	34				
	• Sometimes	50				
**How often do you receive brand name specified prescription?**						
	• Always	68				
	• Never	1				
	• Sometimes	31				
**Do you have any doctor asking to keep aside stock of any specific medicine for incentive purpose?**						
	• No	79				
**Do you check expiry date of medicines before selling to customers?**						
	• Always	93				
	• Never	1				
	• Sometimes	6				
**In which areas do unfair trade practice occur the most?**						
	• Narcotics	2.19				
	• Antimicrobials	2.66				
	• Vitamins/supplements	3.04				
	• Operation theatre products	3.08				
	• General pharmaceuticals	3.29				
	• Gynecological products	3.93				
	• Cardiac agents	4.34				

### Specific demand for antimicrobials

Overall response rate for this domain was 99%. The respondents were also asked about the most common types of clinical presentations for which they were requested to provide antimicrobials. Clinical symptoms indicative of respiratory tract infection was identified as the most prevalent complaint associated with a request for antimicrobials with mean rank of 1.5 (Table 3).

### Characteristics on prescription and sale of antimicrobials

To understand the most frequent types of prescription antimicrobials consumed in Kathmandu Nepal, the respondents were asked questions to identify the most frequent antimicrobials prescribed by doctors by broader infection types. Forty six percent of pharmacy employees ranked azithromycin as the top prescribed antimicrobials for respiratory tract infections. Cefixime was identified by eight percent of respondents as the fourth most prescribed antimicrobial for respiratory tract infections (Table 4). For dental infections, amoxicillin was predominantly identified as the most prescribed antimicrobial by 80% of respondents. For urinary tract infections, different antimicrobial agents of fluoroquinolone family were identified as the most prescribed agents by 80% of respondents. There was though no clear distinction on prescription of specific agent. For other infections related to ear-nose-throat (ENT) and gastrointestinal tract, no clear infection-specific distinction was observed in terms of antimicrobial prescription. For skin infections, though azithromycin, doxycine, cloxacillin and clindamycin were indicated to be the top four most prescribed antimicrobials by half of respondents, other numerous antimicrobials were named by rest half of them. Five common others antimicrobials included cefadroxil (7%), cotrimoxazole (5%), itraconazole (5%), fluconazole (5%), and mupirocin (3%).

**Table 4:** Response of pharmacy employees on the most common antimicrobials prescribed for common infections.

Common infections	The most common antimicrobials prescribed by doctors for respective infections indicated as % responses				
Respiratory tract infections	Azithromycin	Amoxicillin	Amoxicillin-clavulanate	Cefixime	Others
	46	26	13	8	7
Urinary tract infections	Ciprofloxacin	Ofloxacin	Nitrofurantoin	Norfloxacin	Others
	36	23	8	13	20
Ear-nose-throat infections	Amoxicillin	Azithromycin	Ciprofloxacin	Amoxicillin-clavulanate	Others
	28	17	17	15	23
Dental infections	Amoxicillin	Metronidazole	Amoxicillin-clavulanate	Cloxacillin	Others
	80	10	6	0.5	3
Gastro-intestinal tract infections	Metronidazole	Cefixime	Ciprofloxacin	Ofloxacin	Others
	30	21	18	7	24
Skin infections	Azithromycin	Doxycycline	Cloxacillin	Clindamycin	Others
	18	17	9	7	49

### Irrational prescribing behavior

Response rate for this domain was 89%. Fifty eight percent of respondents replied that they had faced problems of irrational prescribing by doctors. Both incorrect dosing and poly-pharmacy were equally identified as irrational prescribing types as observed by 48% of respondents (Table 3). Of other types, unclear handwriting in prescriptions was identified as the most prevalent irrational behavior.

### Practices to tackle irrational prescribing and challenges faced

Overall response rate for this domain was 58%. Asking patients to refer to respective doctor to get a clarification on prescription was ranked as the most prevalent practice to tackle irrational prescribing with a mean rank of 1.76 (Table 3). In contrast, dispensing medicines right away without taking any corrective actions despite knowing that it was not correct, was identified as the least preferred practice with a mean rank of 3.93. Regarding challenges faced by the employees, uncooperative behavior of doctors was ranked as the first most common hurdle faced by 59% of respondents.

### Perception on antimicrobial resistance

Overall response rate for this domain was 99%. A majority (87%) of respondents agreed that AMR is a global public health threat (Table 3). When asked about their knowledge on the factors contributing to AMR, misuse and overuse of antimicrobials was identified as their first most important perception with a mean rank of 1.93. Inadequate sanitary condition was ranked as the least important cause triggering AMR with mean rank of 6.32.

### Efforts on fighting against antimicrobial resistance

Response rate for this domain was 99%. Counseling of patients about prudent use of antimicrobials was ranked as the most important part to be played by the respondents as pharmacists to control AMR with mean rank of 2.36. Discouraging unethical pharmaceutical marketing was perceived as the least important way to fight against AMR with mean rank of 4.39 (Table 3). Additionally, respondents were also asked about their own personal attitude towards use of antimicrobials; 64% of participants responded that they had bought antimicrobials without prescription for their own personal use.

### Unfair trade practice of antimicrobial agents

Overall response rate for this domain was 36%. When asked if they have ever observed unethical commercial practices in their pharmacy, 87% of pharmacy employees agreed on this (Table 3). Fifty nine percent of respondents answered that malpractice was equally prevalent in both government-and privately-owned pharmacies. Forty nine percent of respondents ranked bonus provided by specific pharmaceutical companies to entice sale of specific brands of antimicrobials was the most common malpractice they have observed. When respondents were asked if they ever advised clients to buy a specific brand of medicine, half of them answered that they sometimes did so. In response to the question regarding demand for a specific brand of antimicrobials, 68% of pharmacy employees replied that they always received prescriptions stipulating a specific brand name of antimicrobials; while just 1% of respondents denied this activity.

## Discussion

In this cross-sectional survey conducted among 801 pharmacy employees in LMC of Kathmandu, Nepal, it was evident that inappropriate and irrational prescription of antimicrobial agents is common and widespread in this setting. In high-income countries, such as the United States and those in northern Europe, use of antimicrobials among outpatients is rigorously regulated with strict prescription based dispensing only [6]. However, use of non-prescription antimicrobials is common in most parts of the world [6]. In fact, in some LMICs, non-prescription antimicrobials are the most commonly sold medicines among prescription-only drugs [7]. In our study, most (92%) pharmacy employees agreed that patients asking for antimicrobials without medical prescriptions was a common situation, which is comparable to a study from Eastern Nepal where 77% of respondents agreed on this [5]. This observation is in contrary to the legislation in Nepal that mandates a strict authorized medical prescription for dispensing of antimicrobial agents, thereby revealing non-stringent pharmaceutical regulations in Nepal. A systematic review that included 50 studies conducted in 28 developing countries around the world reported that proportion of antimicrobials dispensed without prescription by pharmacies varied widely, ranging from 3.8% in Tanzania to 100% in Sri Lanka [8].

In LMICs, role of pharmacies extends beyond dispensing medicines as per stipulated medical advice. Pharmacies are also engaged in dispensing OTC non-prescription antimicrobials as per patients’ requests. Not just limited to this, pharmacy operators may even provide their own presumptive diagnosis for patients seeking healthcare, and illegally dispense medicines (including antimicrobials) based on their own limited knowledge or experience. Therefore, pharmacies may play a central role, not only in potentially accelerating local burden of AMR by directly facilitating inappropriate use of antimicrobials, but also by further jeopardizing the health and safety of general public. Therefore, understanding knowledge, attitude, and practice of pharmacy operators on dispensing antimicrobials is of prime importance. Here, 22% (178/801) of pharmacy operators had neither pharmacy specific education nor training. Previous studies from Nepal (36%, 116/321) and India (75%, 18/24) have also reported on involvement of unlicensed practitioners in pharmacies, which may directly impact their dispensing practices [5,9]. In our study, 36% of pharmacy respondents agreed that they dispensed non-prescription antimicrobials, which again corresponds with the earlier survey from Nepal [5]. A higher percentage of pharmacy dispensers from India (67%; 174/261) [10] and Pakistan (97%; 342/353) [11] have also reported to have dispensed non-prescription antimicrobials with or without clarification. In LMICs in South Asia, because of non-stringent government regulations, pharmacies following professional virtue may face commercial disadvantage. Sixty three percent of pharmacy employees in a study from Nepal believed that refusal to dispense non-prescription antimicrobials would lead to a commercial loss, and nearly 90% agreed that patients would somehow obtain non-prescription antimicrobials by attending an alternative pharmacy [5]. Facts such as these might motivate pharmacy employees to deviate from their professional morals. In our study, only half of the respondents perceived that inappropriate use of antimicrobials was the first important factors driving AMR. Earlier study from Nepal reported that nearly 25% of pharmacy respondents declined that inappropriate use of antimicrobials might facilitate AMR [5]. The extent of knowledge, education, and practice as evidenced in this, and other studies from South Asia reflects into the scenario of highly prevalent inappropriate use of antimicrobials in community.

Acute upper respiratory infections (ARTIs) are considered to be the most common diagnosis for prescription of antimicrobials [12]. As viruses are the most common etiological agents, ARTIs are also associated with an increased burden of inappropriate antimicrobials use and subsequent AMR [12,13]. In our study, respiratory tract infection was ranked as the most common complaints for patients requesting antimicrobials with mean rank of 1.50, followed by gastrointestinal (2.53) and urinary infections (2.88). Other study from Nepal also reported that the highest proportions of antimicrobial dispensing were for respiratory and gastrointestinal infections [14]. A study conducted in India reported that 71% (82/115) of pharmacies dispensed antimicrobials without any prescription to the simulated patients presenting with the signs of ARTIs, with 63% (92/115) dispensing antimicrobials for acute gastroenteritis [10]. Further, a study from India reported that pharmacy employees believed that antimicrobials could treat cold (58%, 10/24), viral infection (87%, 20/23), cough (67%, 16/24), and sore throat (71%, 17/24). Such beliefs and practices among pharmacy dispensers are likely key contributors for inappropriate antimicrobial use in LMICs. In this study, azithromycin was ranked as the first most commonly prescribed antimicrobial for respiratory infections, while ciprofloxacin for urinary infections. Further, azithromycin was also ranked as the first most commonly sold antimicrobials in our study, followed by amoxicillin and cefixime. Koju et al. reported that amoxicillin, azithromycin, amoxicillin-clavulanate, cefixime, and ciprofloxacin were the top five most commercially promoted antimicrobials for ARTIs in community pharmacies of Nepal, which could have a direct influence on an increased prescription and sale of these antimicrobials [13]. Another study from Nepal found that cefixime (38%), amoxicillin (29.3%), ciprofloxacin (13.7%), and azithromycin (8.1%) were the most commonly dispensed antimicrobials by community pharmacies [14]. Studies from India and Pakistan have also reported that azithromycin, ciprofloxacin, and cefixime were the most commonly prescribed or sold antimicrobials [10,11]. Azithromycin, cefixime, and ciprofloxacin belong to the watch group antimicrobials under the WHO AWaRe category of antimicrobials, that have increased risk of rendering resistance or toxicity [15]. The finding that these crucial antimicrobials were the most commercially promoted, prescribed, and sold in LMICs portrays a challenging scenario facilitating an escalated emergence and spread of AMR.

The burden of faeco-orally transmitted infectious diseases, such as typhoid, are reflective of sub-standard public health measures and are highly prevalent in LMICs including Nepal [16]. The emergence, selection, and spread of antimicrobial resistant variants of *Salmonella enteric* serovars Typhi and ParatyphiA have been observed, leading to the subsequent treatment failure [1,2,17–19]. Once easily treatable with the first-line antimicrobials such as amoxicillin and cotrimoxazole, the therapeutic management of typhoid is now more challenging, requiring second- and third-line antimicrobials such as azithromycin and parenteral ceftriaxone [17]. A study from Pakistan reported emergence and spread of extensively drug resistant *S*.Typhi harboring genes conferring AMR to all first and second-line antimicrobials along with ceftriaxone, leaving azithromycin as the only widely available oral antimicrobial for treating typhoid [18]. A recent study from Nepal reported clinical isolates of *S*. Typhi that were resistant to azithromycin and ciprofloxacin [19]. We surmise that rampant consumption of azithromycin, cefixime, and ciprofloxacin, as was inferred by their highest prescription and sale in this study, further corroborates the potential scenario of untreatable infectious diseases such as typhoid [17].

Like many survey-based studies, this study also has several limitations. Despite several efforts to ensure complete response from the participants, not all questions could be answered. A long questionnaire having several multiple-choice options each requiring ranking may have discouraged the participants in responding all questions. Further, unavailability of respondents due to closed outlet or too many customers at the time of interview despite earlier scheduling was another unavoidable challenge faced during the study. Unwillingness to respond despite earlier informed consent, which might be because failed to understand the importance of the study, hesitancy to answer some questions that they perceived might be linked to their business, inherent fear of being exposed of some malpractices and fear of inspections were perceived as other factors affecting absolute response from the participants. Designing a simpler questionnaire without ranking questions and composed of fewer concise questions that can be completed in a shorter time in similar survey-based questions may improve response from participants.

## Conclusions

Here, we assessed knowledge, attitude, and practices of pharmacy employees in LMC of Kathmandu, Nepal on various aspects of inappropriate use of antimicrobials. Our study revealed that the demand and without-prescription use of several antimicrobials, including the WHO watch group antimicrobials, is highly prevalent in Kathmandu, Nepal. This scenario may escalate emergence and spread AMR, thereby threatening the therapeutic management of infectious diseases including the otherwise easily treatable ones as evidenced in typhoid fever. Several factors driving inappropriate practice among pharmacy employees were also identified which may aid in formulating and strengthening policies at a local level. Further studies are required to understand perspectives and practices of other functional stakeholders of the community such as prescribing doctors, veterinarians, medicine dealers, policy makers, other concerned authorities, and general population. Results from such will help gather multi-sectorial information on antimicrobial demand, prescription, sale, and consumption to develop holistic strategies to fight against the extant AMR crisis.

## Supporting information

S1 Appendix Antimicrobial Resistance Diaries Questionnaire no. 3. (PDF)

S2 Appendix Raw data. (XLSX)
