# Supplementary material for: Perspectives of pharmacy employees on an inappropriate use of antimicrobials in Kathmandu, Nepal
Source: PLoS One. 2023 May 3;18(5):e0285287. doi: 10.1371/journal.pone.0285287 (PMC10156006; doi:10.1371/journal.pone.0285287)
Supplement: S1 Appendix — (PDF) [file pone.0285287.s001.pdf]

Antimicrobial Resistance Diaries Questionnaire no. 3

Dated:
CODE:

I. Do patients ask for antibiotics without a prescription?

- ☐ Yes
- ☐ No

II. If yes how do you deal with such patients? On a scale of 1-3 with 1 being the highest, please rank the following options according to their occurrence.

- ☐ Ask for prescription
- ☐ Inquire details about indication before dispensing
- ☐ Dispense the antibiotic right away

III. What is the frequency of patients asking antibiotics without a prescription in a day/week/month?

Dated:
CODE:

IV. What are the most commonly prescribed antibiotics by physicians?

A. Respiratory tract infections:
   i)
   ii)
   iii)
B. Urinary Tract Infections:
   i)
   ii)
   iii)
C. ENT infections:
   i)
   ii)
   iii)
D. Skin infections:
   i)
   ii)
   iii)
E. Gastro-enteric diseases(typhoid, diarrhea, dysentery):
   i)
   ii)
   iii)
F. Dental problems:
   i)
   ii)
   iii)

V. Have you noticed any irrational prescribing?

- ☐ Yes
- ☐ No

Dated:
CODE:

VI. If yes kindly elaborate? On a scale of 1-3 with 1 being the highest, please rank the following options according to their occurrence. Please write N/A in non-applicable fields.

- ☐ Wrong Dose
- ☐ Poly pharmacy with more than 1 antibiotics
- ☐ Others:

VII. What is the frequency of irrational prescribing you observed in a day/week/month?

VIII. When faced with irrational prescribing what course of action have you undertaken? On a scale of 1-5 with 1 being the highest, please rank the following options according to their occurrence. Please write N/A in non-applicable fields.

- ☐ Refused to dispense
- ☐ Asked patient to refer to the respective doctor
- ☐ Referred to the respective doctor
- ☐ Dispense anyway
- ☐ Dispense after correction
- ☐ Sought help with a senior pharmacist/ supervisor

Dated:
CODE:

IX. When dealing with irrational prescribing, what are the challenges you had to face? On a scale of 1-3 with 1 being the highest, please rank the following options according to their occurrence. Please write N/A in non-applicable fields.

- ☐ Uncooperative doctors
- ☐ Irritation from the patients
- ☐ Pressure to dispense from employers/ supervisor

X. What are the most common patient complaints involving antibiotics? On a scale of 1-5 with 1 being the highest, please rank the following options according to their occurrence.

- ☐ Respiratory Tract infections
- ☐ Urinary Tract infections
- ☐ Gastro-enteric diseases
- ☐ Skin infections
- ☐ Dental problems

XI. Regarding Lower Respiratory Tract Infections, in which of the following indications have you dispensed antibiotics?

- ☐ Tightness at chest
- ☐ Breathlessness
- ☐ Cough with sputum
- ☐ Wheezing
- ☐ Difficulty in breathing
- ☐ Others

Dated:
CODE:

XII. Regarding Upper Respiratory Tract Infections, in which of the following indications have you dispensed antibiotics?

- ☐ Swollen tonsils
- ☐ Rhinorrhea/blocked nose
- ☐ Discomfort/difficulty in swallowing
- ☐ Red swollen tonsils with pus filled spots
- ☐ high fever
- ☐ cough
- ☐ Others

XIII. Regarding UTI, in which of the following indications have you dispensed antibiotics?

- ☐ Urinary urgency
- ☐ frequent urination
- ☐ flank pain
- ☐ High fever
- ☐ Previous UTI
- ☐ Burning urination
- ☐ Vomiting
- ☐ Others

XIV. Regarding Gastro-enteric diseases, in which of the following indications have you dispensed antibiotics?

- ☐ Abdominal pain
- ☐ more than 6 episodes of diarrhea in the past 24 hours
- ☐ watery diarrhea and persistent vomiting
- ☐ Bloody diarrhea
- ☐ Fever
- ☐ Mucus/blood in stool
- ☐ Few episodes of loose stool
- ☐ Symptoms of dehydration
- ☐ Others

Dated:
CODE:

XV. What do you understand by antimicrobial resistance?

- ☐ Antimicrobial resistance occurs when microorganisms undergo genetic changes which cause them to become resistant to antimicrobials.
- ☐ An antibiotic becomes resistant when it is ineffective towards a microorganism which was previously susceptible to it.
- ☐ Antimicrobial resistance is a condition in which the microorganisms become resistant and antibiotic fails to show efficacy towards it which requires the use of broad-spectrum antibiotics.
- ☐ I do not know

XVI. What kind of activities might trigger/ cause antibiotic resistance? On a scale of 1-8 with 1 being the highest, please rank the following options. Please write "does not know" in non-applicable fields.

- ☐ Misuse and overuse of antimicrobials
- ☐ Irrational prescribing
- ☐ Poor patient compliance
- ☐ Genetic changes of microorganisms
- ☐ Poor infection control
- ☐ Inadequate sanitary conditions
- ☐ Uncontrolled over-the-counter sale of antibiotics
- ☐ Unregulated antibiotic marketing

Dated:
CODE:

XVII. Do you consider antimicrobial resistance as a global threat?

- ☐ Yes
- ☐ No

XVIII. How serious is this issue with regards to our community?

- ☐ Not serious
- ☐ Somewhat serious
- ☐ Serious
- ☐ Very serious
- ☐ Extremely serious

XIX. What can be done from your part as pharmacists to control antimicrobial resistance? On a scale of 1-5 with 1 being the highest, please rank the following options.

- ☐ Judicious dispensing of antimicrobials
- ☐ Patient counseling regarding prudent use of antimicrobials
- ☐ Improve patient compliance by extensive counseling
- ☐ Discourage unethical pharma marketing
- ☐ Communicate with physicians for better patient treatment

Dated:
CODE:

XX. What can be done to increase pharmacist-doctor collaboration to combat AMR?

XXI. What are the bestselling antimicrobials in your establishment along with their indications?

1.
2.
3.

XXII. What are the Top 3 selling broad spectrum antibiotics?

1.
2.
3.

Dated:
CODE:

XXIII. What kind of challenges do you face while dispensing antimicrobials? On a scale of 1-4 with 1 being the highest, please rank the following options according to their occurrence.

A. Patient-specific:
- ☐ Self-medication
- ☐ Patients unwilling to participate in counseling
- ☐ Patients asking for higher end antibiotics
- ☐ Less aware and uneducated patients

B. Treatment-specific:
- ☐ Irrational prescribing
- ☐ Poly pharmacy
- ☐ Overuse of antibiotic
- ☐ Frequent use of higher end antibiotics

XXIV. What are your perception regarding patient's knowledge and attitude regarding AMR?

XXV. Have you ever bought drugs from a pharmacy?
- ☐ Yes
- ☐ No

XXVI. Have you ever bought antibiotics from a pharmacy without a prescription?
- ☐ Yes
- ☐ No

Dated:
CODE:

XXVII. When was the last time you bought antibiotics for personal use?

- ☐ In the last month
- ☐ In the last 6 month
- ☐ In the last year
- ☐ More than 01 year
- ☐ Don't remember

XXVIII. Have you ever stored antibiotics at home for future use?

- ☐ Yes
- ☐ No

XXIX. Have you noticed any unfair trade practices in your career?

- ☐ Yes
- ☐ No

XXX. What are the common types of unfair trade practices you have noticed in your practice? Please rank in order, with 1 being the highest. 0=N/A

- ➢ ☐ False representation
- ➢ ☐ Free gifts
- ➢ ☐ Bonus
- ➢ ☐ Free samples

XXXI. Do pharmaceutical companies offer sample medicine to you?

- ☐ Yes
- ☐ No

Dated:
CODE:

XXXII. Do you give advice to patients to purchase a particular brand's medicine?

- ☐ Always
- ☐ Sometimes
- ☐ Never

XXXIII. How often do you receive prescription in brand name?

- ☐ Always
- ☐ Sometimes
- ☐ Never

XXXIV. If you do not have the prescribed brand name, do you offer/substitute another medicine containing same composition?

- ☐ Always
- ☐ Sometimes
- ☐ Never

XXXV. Does any company offer free gifts to sell their medicine?

- ☐ Always
- ☐ Sometimes
- ☐ Never

XXXVI. Do you check expiry date of medicines before selling to the customers?

- ☐ Always
- ☐ Sometimes
- ☐ Never

Dated:
CODE:

XXXVII. Do you have any doctor asking to keep aside the stock of any specific medicine for their commission?

- ☐ Yes
- ☐ No

XXXVIII. In which area does the unfair trade practice occur more?

- ☐ Government
- ☐ Private
- ☐ Both are equal

XXXIX. In which area do unfair trade practice occur n higher amount? Rank in order with 1 being the highest and 0=N/A

- ☐ Operation theatre products
- ☐ Gynecological products
- ☐ Antimicrobials
- ☐ Narcotics
- ☐ Cardiac agents
- ☐ Vitamins/supplements
- ☐ General pharmaceuticals

XL. Name some companies who offer free gifts after getting demand orders.
